# Relationship between the level of physical activity and body mass index to blood pressure among overweight and obese young adults in the Northern Emirates city: A cross-sectional study

**DOI:** 10.1371/journal.pone.0304360

**Published:** 2024-06-20

**Authors:** Naina Choudhary, Kumaraguruparan Gopal, Waqar Naqvi, Praveen Kumar Kandakurti, Animesh Hazari

**Affiliations:** Department of Physiotherapy, College of Health Sciences, Gulf Medical University, Ajman, UAE; University of Sharjah, UNITED ARAB EMIRATES

## Abstract

**Background:**

Obesity affects both adults and children all over the world and it is a major causative factor for diabetes, cardiovascular disease, different types of cancer, and even death. Therefore, this study aimed to assess the level of PA and BMI to the risk of developing high BP among overweight and obese young adults.

**Methodology:**

A cross-sectional study was carried out in the Thumbay Medi-city Northern Emirates, Ajman, UAE. Participants enrolled in the study under the convenient sampling method and inclusion criteria: young overweight and obese individuals, male and female, aged between 18 to 30 years. Approval was obtained from the Institutional Review Board (CoHS, GMU (IRB-COHS-STD-110-JUNE-2023). The blood pressure and body mass index were clinically measured using standard tools whereas the GPAQ questionnaire was used to determine the level of physical activity of all participants.

**Results:**

Out of 206 participants, 139 were overweight and 67 were obese. Further, 89 were found to have high normal BP, 93 normal BP, and 24 were found to have optimal blood pressure. The mean GPA scores were 322.8±62.28 in overweight individuals and 301.17±49.05 in obese individuals. Furthermore, among overweight and obese participants there is a weak correlation between PA & BMI (r = 0.06, p = 0.88) and (r = 0.15, p = 0.44) and the BP and BMI (r = 0.18, p = 1.02) and (r = 0.16, p = 0.90) were found.

**Conclusion:**

Although PA, BMI, and BP are assumed to be related variables leading to various non-communicable diseases the present study showed a weak correlation between the level of PA and BMI to the risk of developing BP among overweight and obese young adults in the Northern Emirates.

## Introduction

Obesity is defined as, “abnormal or excessive fat accumulation that may impair health significantly [1]. This is a major causative risk factor of other disorders including diabetes, cardiovascular disease, and different types of cancer leading to markedly diminished life expectancy [2]. Recent years have seen a significant rise in the prevalence of overweight children, adolescents, and adults worldwide, not just in high-income nations but also in less-developed ones [3]. Gaining weight is linked to an increase in heart rate and a decrease in blood vessel flexibility. Extra calories are stored as fat in the body’s fatty tissues. The body needs more oxygen and nutrients due to this fatty tissue, which raises blood circulation levels. Increased blood flow through arteries results in higher pressure [4].

The United Arab Emirates (UAE) has experienced a rapid epidemiological shift from a traditional semi-nomadic civilization to a modern affluent society with a lifestyle defined by excessive eating of foods high in energy with performance of little physical activity [5–7]. Cardiovascular disease (CVD) accounts for more than a quarter (29%) of all deaths in the UAE [8] with a high prevalence of cardio-metabolic risk factors [9]. According to recent data, more than 65% of individuals are overweight or obese, with 57% having central obesity [10]. The American Diabetes Association’s standards report that the rate of metabolic disorder in the UAE is significantly greater than that of the United States (US), with the highest obesity burden (44%) [11].

One of the main factors contributing to overweight and obesity is poor physical activity levels. It is commonly acknowledged that reduced physical activity leads to obesity and in turn, causes CVDs. A study has shown that a high incidence of CVD, including coronary heart disease and stroke, is associated with being overweight or obese [12]. Fundamentally, obesity may cause heart failure (HF) by altering hemodynamic parameters associated with renin-angiotensin-aldosterone system activation, enhancing the activity of the sympathetic nervous system and mineralocorticoid receptor expression, and producing acute-phase proteins and inflammatory cytokines [13]. Mechanically, vascular smooth muscle cell proliferation, endothelial dysfunction brought on by obesity, or increased sympathetic nervous activity cause arterial stiffness, which leads to hypertension. In addition, age-related changes in body composition result in an increase in the waist-hip ratio (WHR) with a loss of muscle mass and strength due to the redistribution of fat from peripheral and subcutaneous sources to a central location.

In addition, a poor level of cardiorespiratory fitness is a powerful clinical predictor of CVD, cardiovascular disease mortality, and all-cause mortality [14]. It is well known that Physical Activity has an important role in combating morbidities related to cardiovascular disorders and non-communicable diseases. However, there is a dearth of literature establishing this relationship in the United Arab Emirates population. There is limited data on physical activity and its relationship with lifestyle disease in young adults in the UAE. Modernization and industrialization have led to a change in lifestyle of young adults which increases the risk for lifestyle diseases like hypertension, obesity, and diabetes mellitus [6]. A significantly higher prevalence of diabetes and hypertension is reported in the Northern Emirates [7]. The current young population leading a sedentary lifestyle may have a direct effect on their level of physical activity, BMI, and systolic and diastolic blood pressure (SBP and DBP). The growing literature on the relationship between physical activity, BMI, and BP in overweight and obese individuals, fails to fill the significant population gap in the existing research on young adults living in the northern emirate city, Ajman UAE. Research on the relationship between physical activity, BMI, and BP in this population is lacking. Hence, there is a need for early screening in young individuals for physical activity, BP, and BMI as this would provide direction for early lifestyle modification.

Therefore, this study evaluated the relationship between the level of physical activity and BMI to the risk of developing BP among overweight and obese young adults in the Northern Emirates city. The objectives of the research were as follows:

1. To evaluate the relationship between the level of physical activity and blood pressure among overweight and obese young adults in the Northern Emirates.
2. To evaluate the relationship between body mass index and blood pressure among overweight and obese young adults in the Northern Emirates.
3. To study the prevalence of level of BP, levels of PA and BMI among young adults in the Northern Emirates.

## Methodology

### Research design and settings

A cross-sectional study was conducted Department of Physiotherapy, Thumbay Physical Therapy and Rehabilitation Center, Thumbay University Hospital, Gulf Medical University, Ajman, United Arab Emirates.

### Study population and sampling

Participants enrolled in the study under the convenient sampling method and inclusion criteria: young overweight and obese individuals, male and female, aged between 18 to 30 years. Participants with acute illness and active pregnancy were excluded from the study. A total of 216 participants enrolled in the study who met the inclusion criteria after initial screening of 837 unknown participants. The sample size calculation was done for a 95% Confidence Interval and precision set as.05 based on the overall prevalence of 17% for obesity in the United Arab Emirates conducted by a recent study [15].

### Study instruments

A standardized and validated digital sphygmomanometer (OMORON M7 BP monitor) with high specificity (93.8%) and sensitivity (95.5%) [16], Stadiometer with high degree of reliability of Inter-correlation coefficient (ICC = 0.999) [17], and Global Physical Activity Questionnaire (GPAQ) with reliability ranging from good to very good (r = 0.58–0.89) [18] was used for the present study.

### Study procedure

An informed written informed consent was obtained from all participants. Ethical approval for the study was obtained from the Institutional Research Board (IRB) IRB-COHS-STD-110-JUNE-2023, College of Health Sciences, Gulf Medical University. The data recruitment started from July 3^rd^, 2023, till January 5^th^ 2024. Each subject provided demographic information, such as age, gender, and occupation. Blood Pressure was measured by using a digital sphygmomanometer (OMORON M7 BP monitor) and Physical Activity was measured by using the Global Physical Activity Questionnaire (GPAQ) [19]. Body weight was measured with the participant wearing light clothing on an electronic scale, height in centimeters with a stadiometer, and BMI denoted as weight/height^2^ (kg/m2). All variables were measured by the same physical therapist for all participants using standard clinical procedures.

### Data storage and analysis

The raw data was extracted from the individual data sheet and stored in the form of Google Sheets and later analyzed using the statistical software package SPSS 22. Upon analysis, the data was stored in the drive of the researcher. The data was collected from everyone separately via personal email and password-protected for security. Descriptive analysis was used in terms of Frequencies and percentages for the calculation of demographic data. The test of normality was conducted before inferential stats. The data was not normally distributed and thus the non-parametric tests were used. The continuous variables were measured using the mean and standard deviation. The Spearman’s rank correlation coefficient was used to analyze the relationship between Physical activity and blood pressure and body mass Index with Blood Pressure. There were 10 missing data of more than 20 percent of the input and thus removed from the data analysis.

## Results

**Fig 1:**
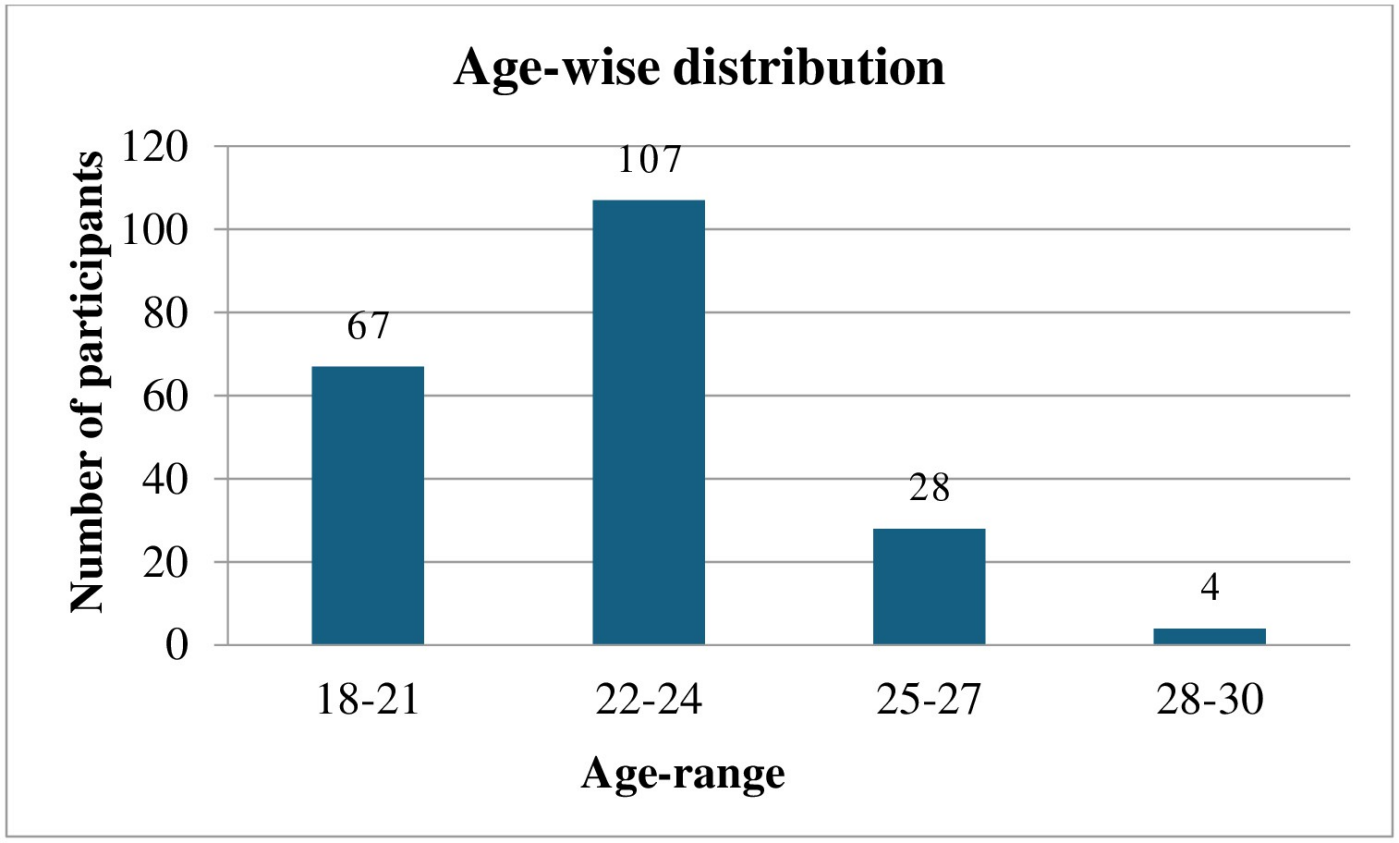
Age-wise distribution of participants.

**Fig 2:**
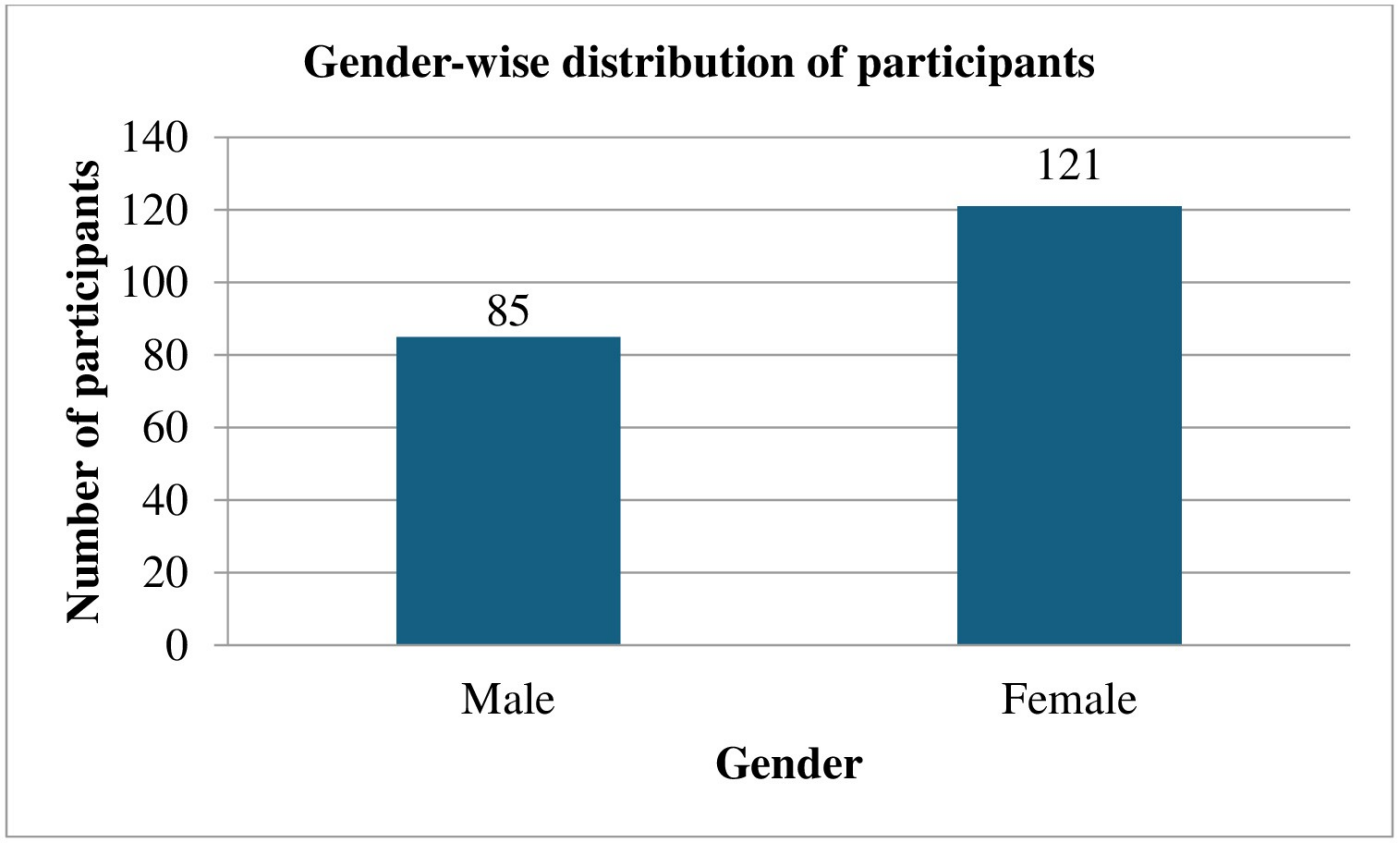
Gender-wise distribution of participants.

**Fig 3:**
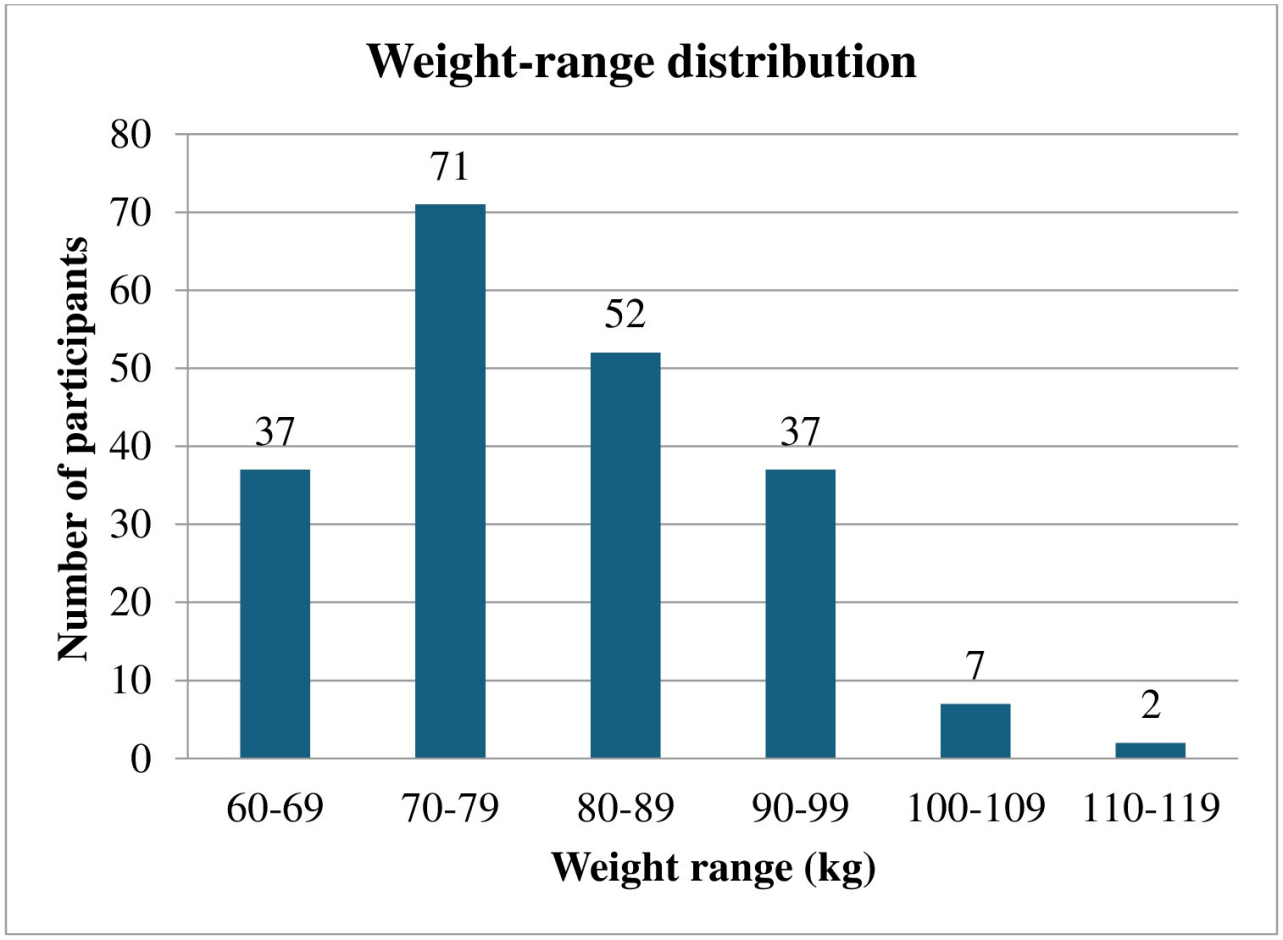
Weight range distribution of participants.

Overall, 206 participants’ data was analyzed out of 216, consisting of overweight and obese populations within the age range of 18–30 years as shown in Fig 1. Gender-wise and weight-range distribution is shown in Figs 2 and 3.

Maximum participants were in the age group between 22–24 years consisting of 107 (51.94%) participants, followed by 18–21 years with 67 (32.52%) participants, 25–27 years with 28 (13.59%) participants, and 28–30 years with 4 (1.94%) participants.

Graph 2 demonstrates that out of 206 samples, the maximum number of participants were females consisting of 121 (58.74%) and 85 (41.26%) were males.

Out of 206 samples, maximum participants were in the weight range between 70–79 kg consisting of 71 (34.46%) participants, followed by 52 (25.24%) participants in the weight range between 80–89 kg, 37 (17.96%) participants each in the weight range between 60–69 kg and 90–99 kg, 7 (3.39%) participants in the weight range between 100–109 kg, 2 (0.97%) participants in the weight range between 110–119 kg.

Out of 206 samples, 139 (67.48%) participants were overweight, and 67 (32.52%) participants were obese (Table 1).

**Table 1:** Distribution of participants in overweight and obese categorization and means of BMI.

Body Mass Index (BMI) based Classification	Observations	BMI Mean±SD
Overweight	139 (67.48%)	27.37±1.53
Obese	67 (32.52%)	31.64±1.86
**Total**	206 (100.00%)	

Out of 206 samples, 89 (43.2%) participants were found to have high normal BP, 93 (45.1%) participants were found to have normal BP values, and 24 (11.6%) participants were found to have optimal BP values based on WHO blood pressure classification (Table 2).

**Table 2:** Distribution of participants based on BP (Mean± S.D).

Blood Pressure (mmHg)	Number of participants (%)	Systolic B.P Mean±SD	Diastolic B.P Mean±SD
Optimal	24 (11.6%)	114.08±3.7	80.4±6.5
Normal	93 (45.1%)	125.6±2.7	88.5±5.1
High normal	89 (43.2%)	133.5±2.8	89.9±3.6

The physical Activity (PA) measurement of participants is illustrated in Table 3. Out of 206 samples, 139 (67.48%) consisted of overweight individuals and 67 (32.52%) obese individuals. The mean GPAQ scores were found to be 322.8±62.28 in overweight individuals and 301.17±49.05 in obese individuals.

**Table 3:** Distribution of participants based on physical activity questionnaire.

Body Mass Index (BMI)	Observations	Global Physical Activity Questionnaire (GPAQ) Mean±SD
Overweight	139 (67.48%)	322.8±62.28
Obese	67 (32.52%)	301.17±49.05
**Total**	206 (100.00%)	

The correlation between PA and BP is demonstrated in Table 4. The Spearman’s RANK correlation coefficient (r-value) for overweight subjects was found to be 0.067 and the p-value was found to be 0.88 whereas, the r-value was found to be 0.157 and the p-value was found to be 0.44 in obese participants. There is a weak association of RANK between PA and BMI and the obtained p-value showed statistically insignificant.

**Table 4:** Correlation between physical activity and body mass index (N = 206).

**Global Physical Activity Questionnaire (GPAQ)**	**Blood pressure**		
		r-value	p-value
	Overweight	0.06	0.88
	Obese	0.15	0.44

The correlation between BMI and BP is demonstrated in Table 5. The correlation coefficient (r-value) for BP in overweight participants was found to be 0.187 and the p-value was found to be 1.02 whereas in obese participants the r-value was 0.167 and the p-value was found to be 0. 90. The Spearman’s RANK correlation takes a value from -1 to +1 therefore, there was a weak association of RANK between BMI and BP. The obtained p-value was more than 0.05 which suggests statistically insignificant findings.

**Table 5:** Correlation between body mass index and blood pressure.

**Blood Pressure (mmHg)**	**Body Mass Index (BMI) (N = 206)**		
		r-value	p-value
	Overweight	0.18	1.02
	Obese	0.16	0.90

## Discussion

The goal of the study was to determine the relationship between the level of PA and BMI to the risk of developing BP in individuals in the Northern Emirate cities who were overweight or obese. The gender-wise distribution of participants demonstrated that the maximum number of participants were females consisting of 121 (58.74%) and 85 (41.26%) were males (Graph 2). AL-Shamsi S et al. examined the incidence of CVD and the risk variables that are linked to it in a retrospective cohort research in men and women and reported that the maximum number of participants found were females consisting of 492 participants and males 485 out of a total 977 participants [8]. In addition to this study, a prior study found that 43 participants were boys and 63 participants were girls and the majority of them were overweight or obese [1]. A study carried out in South India revealed a similar conclusion that stated girls were more likely than boys to be overweight or obese [20]. Another study conducted in Tanzania found that girls (26.7%) were more likely than boys (17%) to be overweight or obese [21]. Similarly in Eastern Ethiopian research, girls (20.90%) had higher rates of overweight and obesity than boys (20.30%), though the difference was not statistically significant [22]. This could be because females tend to gain more weight during adolescence. It is accepted that girls are more prone than boys to become overweight and obese during puberty due to physiological weight gain during this time [23].

The weight range distribution of participants reported that out of 206 samples, the maximum participants were in the weight range between 70–79 kg consisting of 71 (34.46%) participants, followed by 52 (25.24%) participants in the weight range between 80–89 kg, 37 (17.96%) participants each in the weight range between 60–69 kg and 90–99 kg, 7 (3.39%) participants in the weight range between 100–109 kg, 2 (0.97%) participants in the weight range between 110–119 kg (Graph 3). Additionally, classification based on BMI into overweight and obese reported that out of 206 samples, 139 (67.48%) participants were overweight, and 67 (32.52%) participants were found to be obese. According to a prior study presented by Lemamsha H., there were 42.4%, 32.9%, and 24.7% of Libyan individuals who were obese, overweight, or of normal weight, respectively [24]. According to the results, roughly 75.3% of adults in Libya were overweight or obese [24]. According to a survey conducted by Al Sabbah H, of the total, 71.7% indicated that they had irregular meals [25]. Obese (89.3%) and overweight (78.0%) students reported having irregular meals at higher percentages than students who were normal weight (65.4%) [25]. The main contributing factors included leading a sedentary lifestyle, urbanization, moving from rural to urban areas, eating meals high in energy, and not exercising. Among the several indicators, oxidative stress, microbiota, adipocytes, and microRNAs showed promising results in identifying obesity [26].

### Correlation between PA and BMI

According to a recent study presented by Dalibalta S et al., young adults in the UAE have high levels of sedentary lifestyle, and PA is primarily light intensity [27]. As a result, both PA and sedentary behaviour should be closely monitored across various cohorts in this nation. When compared to young UAE males, young UAE females appear to be engaging in less moderate-to-vigorous intensity physical activity (MVPA) and daily step counts. This suggests that researchers and healthcare professionals should create targeted interventions that promote higher-intensity activity in young UAE females [27]. The result from the present study suggests that overweight individuals were found physically active in comparison to obese individuals as per the WHO recommendations for MET minutes/week [28]. The overall correlation between PA with BMI was found to be statistically insignificant (Table 4) suggesting a failure to establish a statistical correlation although clinical correlation could be present. An in-depth understanding of how PA affects weight reduction is provided by some recent systematic reviews of the research. To enumerate the impact of PA on changes in body weight, the Advisory Committee for the 2008 Physical Activity Guidelines for Americans examined the existing scientific literature [28]. The review’s findings indicated that engaging in moderate-to-intense physical exercise for at least 150 minutes a week—such as brisk walking—would result in a 1%–3% decrease in body weight [28]. Studies in the past have shown that aerobic exercise interventions raise high-density lipoprotein and decrease triglycerides. For this reason, fitness training helps in weight management for those who are obese with dyslipidemia [29].

However, without decreased energy calory intake, PA can have little effect on body weight loss as the extent of the reduction varies according to the amount of PA the individual is engaged in. It is generally acknowledged that the most effective lifestyle therapies for overweight and obese people involve combining a reduction in caloric intake with an increase in PA. In addition to the weight reduction resulting from energy restriction alone, PA also helps with weight loss [30]. It is therefore imperative that clinicians understand the role that good eating habits and sufficient PA are essential for long-term weight loss in adults who are overweight or obese. When it comes to the participation of overweight and obese people in PA, barrier identification appears to be yet another crucial issue that needs to be considered. Adults who are overweight or obese have barriers to PA which have been previously reported and the most frequently mentioned barriers are a lack of time and enthusiasm. Self-reported barriers to PA appear to be reduced by traditional behavioural weight loss programs that include both diet and PA. Therefore, health professionals should ask young adults who are overweight or obese about the obstacles they experience in their way of engaging in PA, and then use their problem-solving abilities to come up with appropriate and feasible solutions [28].

### Correlation of BMI and BP

Previous studies suggest that both obesity and BMI are risk factors for hypertension [31]. Furthermore, a greater BMI percentile is linked to higher blood pressure. In consensus, a cross-sectional population-representative study conducted by Al Junaibi A et al. determined the prevalence and drivers of obesity in childhood and adolescence and their association with BP [32]. In the present study, the correlation coefficient (r-value) for BP in overweight participants and obese participants suggested a weak correlation between BMI and BP as the p-value was more than 0.05, found to be statistically insignificant (Table 5). Thus, it could be conferred that although BMI and BP were clinically correlated, they failed to establish a stronger statistical significance due to possible interactions of factors affecting both BMI and BP. However, the study conducted by Al Junaibi A et al. concluded that the BMI-BP association became stronger with time and higher percentile for obesity among school children and adolescents in Abu Dhabi [32], and could have similar effects on other parts of the Emirates.

Previous studies have observed the opposite trend of increased BMI with decreased BP, in contradiction to the findings. The mean BMI (27.0 kg/m2) in the adult population of Germany did not change, while the prevalence of obesity did slightly rise between 1998 and 2011. However, over time, both the mean SBP and the strength of the BMI-SBP connection declined, from 129.0 to 124.1 mmHg [33]. Similar findings were noted in adults in research conducted between 1989 and 2004. While the prevalence of treated hypertension significantly increased, the mean SBP and DBP in men and women respectively modestly dropped from 133/87 to 131/86 mmHg and 127/82 to 124/81 mmHg. Despite receiving hypertension medication, the mean BMI increased significantly, although the correlation between BMI and blood pressure decreased [34]. In addition, the relationship between BMI and BP may be impacted by variables like levels of PA, dietary sodium, alcohol intake, smoking, and drugs for BP [35–39]. Insufficient vasodilatation in the presence of elevated blood volume and cardiac output, which are natural outcomes of increased mass, could be the root cause of the positive association observed between BMI and BP [30]. In a country like UAE, the above-mentioned risk factor could prevail due to socioeconomic, geographic, and cultural differences attributed to the findings of the study. Further research could be done to explore these factors under a multivariate regression analysis.

### Correlation between PA and BMI to BP

In the present study, results indicated that there is a weak correlation between BMI with BP and Physical activity with BP which was found to be statistically insignificant. However, 89 (43.2%) of the participants are under the high normal category of hypertension and they are more prone to develop stage-1 hypertension as per WHO classification. Also, the overall physical activity as per the GPAQ score did not meet the WHO recommendations on physical activity for health [28]. It is commonly known that PA, BMI, and BP are interrelated. According to estimations, for every 5% rise in body weight, there is a 20–30% greater chance of hypertension, and reduced PA is one of the major causes of increased body weight. Clinical research indicates that weight loss lowers blood pressure in the majority of hypertensive people and that maintaining a BMI < 25 kg/m^2^ is beneficial in the primary prevention of hypertension [40]. Furthermore, engaging in vigorous PA that improves cardiorespiratory fitness can help lower health risks in individuals who are overweight or obese, regardless of the impact of exercise on body weight. Additionally, PA leads to decreased resistance to the vessels, reduced stiffness of the arteries, reduced oxidative stress and inflammation, increased angiogenesis and arteriogenesis, increased arterial compliance and diameter that consequently result in control of BP, and reduction in body weight or body mass leading to control of progression in overweight or obesity [12]. Hence, assessing PA among overweight and obese individuals at regular intervals is essential as a preventive measure for the risk of developing BP.

In the present study, a weaker correlation of PA with BMI and BP suggests that there could be other factors contributing to non-communicable diseases such as obesity and hypertension in the United Arab Emirates. Previous studies have reported that PA has a strong correlation with weight management and blood pressure [12] which doesn’t stand true in our study. This demands further research to explore in the context of the UAE.

## Conclusion

Although PA, BMI, and BP are assumed to be related variables leading to various non-communicable diseases the present study showed a weak correlation between the level of PA and BMI to the risk of developing BP among overweight and obese young adults in the Northern Emirates. PA and BMI have a weak indicator in developing BP but should be related to the Hip Waist Ratio (HWR). There is a need to identify this gap with the contributing factors other than reduced PA for very high prevalence of Obesity, hypertension, and other non-communicable disease like diabetes mellitus.

### Limitations

The cross-sectional study design limited to a small geographical area could be a potential limitation of the study as the findings would lack generalization to the entire Emirates. Although the sample size was calculated, the smaller sample population could again lead to difficulties in the generalization of the findings. Moreover, multiple factors could have affected the BMI, and BP could have been better studied through a multiple logistic regression analysis which was lacking in the present study.

### Future recommendation

A longitudinal study could be replicated in the context of different populations and age groups using a larger sample size. Multiple factors could be explored with a logistic regression analysis. Considering the higher prevalence of obesity and overweight in the Northern Emirates, tailored interventions would be required for future studies.

## Supporting information

S1 File Naina masterchart 206 samples. (XLSX)
